# Long noncoding RNA *FOXD2-AS1* enhances chemotherapeutic resistance of laryngeal squamous cell carcinoma via STAT3 activation

**DOI:** 10.1038/s41419-020-2232-7

**Published:** 2020-01-20

**Authors:** Rui Li, Shuwei Chen, Jiandong Zhan, Xinghua Li, Wenlin Liu, Xiaoli Sheng, Zhongming Lu, Rong Zhong, Liangsi Chen, Xiaoning Luo, Yameng Hu, Ying Ouyang, Tao Liu, Quan Zhang, Siyi Zhang

**Affiliations:** 1 Guangdong Provincial People’s Hospital, Guangdong Academy of Medical Sciences, School of Medicine, South China University of Technology, Guangzhou, China; 2 Department of Otorhinolaryngology, Guangdong Provincial People’s Hospital & Guangdong Academy of Medical Sciences, Guangzhou, China; 3 Department of Head and Neck Surgery, Sun Yat-sen University Cancer Center, Guangzhou, China; 4 State Key Laboratory of Oncology in South China, Guangzhou, China; 5 Collaborative Innovation Center of Cancer Medicine, Guangzhou, China; 6 Department of Radiotherapy, The Affiliated Yantai Yuhuangding Hospital of Qingdao University, Yantai, China; 7 Department of Otorhinolaryngology, The Sixth Affiliated Hospital of Guangzhou Medical University, Qingyuan People’s Hospital, Guangzhou, China; 8 Department of Biochemistry, Zhongshan school of medicine, Sun Yat-sen University, Guangzhou, China; 9 Sun Yat-sen University Cancer Centre, State Key Laboratory of Oncology in Southern China, Collaborative Innovation Centre for Cancer Medicine, Guangzhou, China

**Keywords:** Cancer stem cells, Cancer therapeutic resistance, Head and neck cancer, Predictive markers, Head and neck cancer

## Abstract

Laryngeal squamous cell carcinoma (LSCC) is a common head and neck cancer. Despite recently improved management of LSCC, chemotherapy resistance of patients remains a challenge. In this study, we identified that long noncoding RNA *FOXD2-AS1* regulates LSCC therapeutic resistance by augmenting LSCC stemness. LSCC chemotherapy-resistant patients showed increased *FOXD2-AS1* expression compared with that in chemotherapy-sensitive patients, which predicted poor prognosis. Gain- or loss-of-function experiments showed that upregulated *FOXD2-AS1* maintained cancer stemness, reducing the response to chemotherapy, while *FOXD2-AS1* downregulation had the opposite effects. *FOXD2-AS1* acted as a scaffold for STAT3 and PRMT5, promoting STAT3 transcriptional activity, which is essential to maintain cancer stemness and promote chemotherapeutic resistance. Interfering with *FOXD2-AS1* using short hairpin RNA rescued LSCC’s chemotherapeutic sensitivity. Thus, *FOXD2-AS1* promotes LSCC chemotherapeutic resistance and is an upstream activator of *STAT3*, making *FOXD2-AS1* a potential therapeutic target to improve the chemotherapy effect in LSCC patients.

## Introduction

Laryngeal squamous cell carcinoma (LSCC) is one of the most common subtypes of laryngeal cancers and has the second highest incidence among respiratory tumors^1^. Notably, the occurrence of LSCC in China is approximately four times higher than that in the United States, with >15,000 estimated deaths each year^2^. For patients with late-stage LSCC, chemotherapy is the standard first-line treatment^1,3^, which stops the growth of cancer cells, either by killing the cells or by stopping the cells from dividing. However, platinum-based combined chemotherapy approaches did not induce an overall survival (OS) benefit in patients with advanced LSCC, resulting from toxicity of these agents and chemoresistance of the patients^4^. Therefore, revealing the underlying mechanisms of chemotherapy resistance in LSCC and identifying novel biomarkers for targeted therapy are urgently required to improving the dismal outcome of this disease.

The prevalence of cancer stem cells (CSCs) and the heightened propensity for tumors to maintain CSC subpopulations, also known as cancer stemness, are essential for tumors to establish resistance to therapy^5,6^. Recent studies revealed that reducing CD133^+^ CSC populations in LSCC cells in vitro could elevate the cancer cells’ sensitivity to cisplatin treatment^7–9^. These results not only reflected the impact of reducing CSCs on cancer cell longevity, but also highlighted the substantial effects that CSCs have on enhancing chemoresistance. It is likely that inherent cancer cell drug resistance arises from the tendency of CSCs to display heightened DNA repair, cell quiescence, and the expression of ATP-binding cassette transporters that promote drug efflux^10^. When CSCs survive an initial course of chemotherapy, they can adopt a drug resistance phenotype that contributes to acquired chemoresistance. In laryngeal carcinoma, a number of chemoresistance mechanisms have been identified^9,11–13^, and in this study, we sought to further verify the molecular mechanism of laryngeal carcinoma stemness and chemoresistance.

Recently, emerging evidence indicated that long noncoding RNAs (lncRNAs) participate in tumor progression and drug resistance^14–16^. LncRNAs are a type of RNAs with no or weak protein-coding potential that function mainly by acting as host genes for microRNAs, by serving as molecular scaffolds to guide proteins to their direct chromosomal targets, or by protecting targeted proteins from degradation. Different lncRNAs confer chemoresistance in several types of cancer cells by changing multiple drug resistance transporters, modulating the cellular anti-apoptosis potential, affecting the epithelial mesenchymal transition (EMT) process, and modulating signaling pathways^15^. However, the identification of the lncRNAs that might be involved in LSCC stemness and chemoresistance, and their precise mechanisms remain limited^17^.

In the present study, we found that lncRNA *FOXD2-AS1* was significantly upregulated in patients with advance LSCC. Gain- or loss-of-function experiments showed that lncRNA *FOXD2-AS1* is a key mediator of the development of chemotherapeutic resistance in LSCC, being involved in cancer stemness maintenance. Mechanistically, *FOXD2-AS1* serves as a scaffold of signal transducer and activator of transcription 3 (STAT3) and protein arginine methyltransferase-5 (PRMT5), thereby augmenting the transcriptional activity of STAT3, which is required to maintain CSCs subpopulations in tumors. More importantly, silencing *FOXD2-AS1* using short hairpin RNAs (shRNAs) restored the chemotherapy sensitivity of LSCC cells to a level similar to treatment with stattic, a STAT3-selective inhibitor. Therefore, our results revealed a novel mechanism mediated by *FOXD2-AS1* to maintain the cancer stemness of LSCC, and highlighted the clinical significance of *FOXD2-AS1* in LSCC therapy.

## Results

### FOXD2-AS1 was upregulated in LSCC and correlated with LSCC chemoresistance

**Fig. 1:**
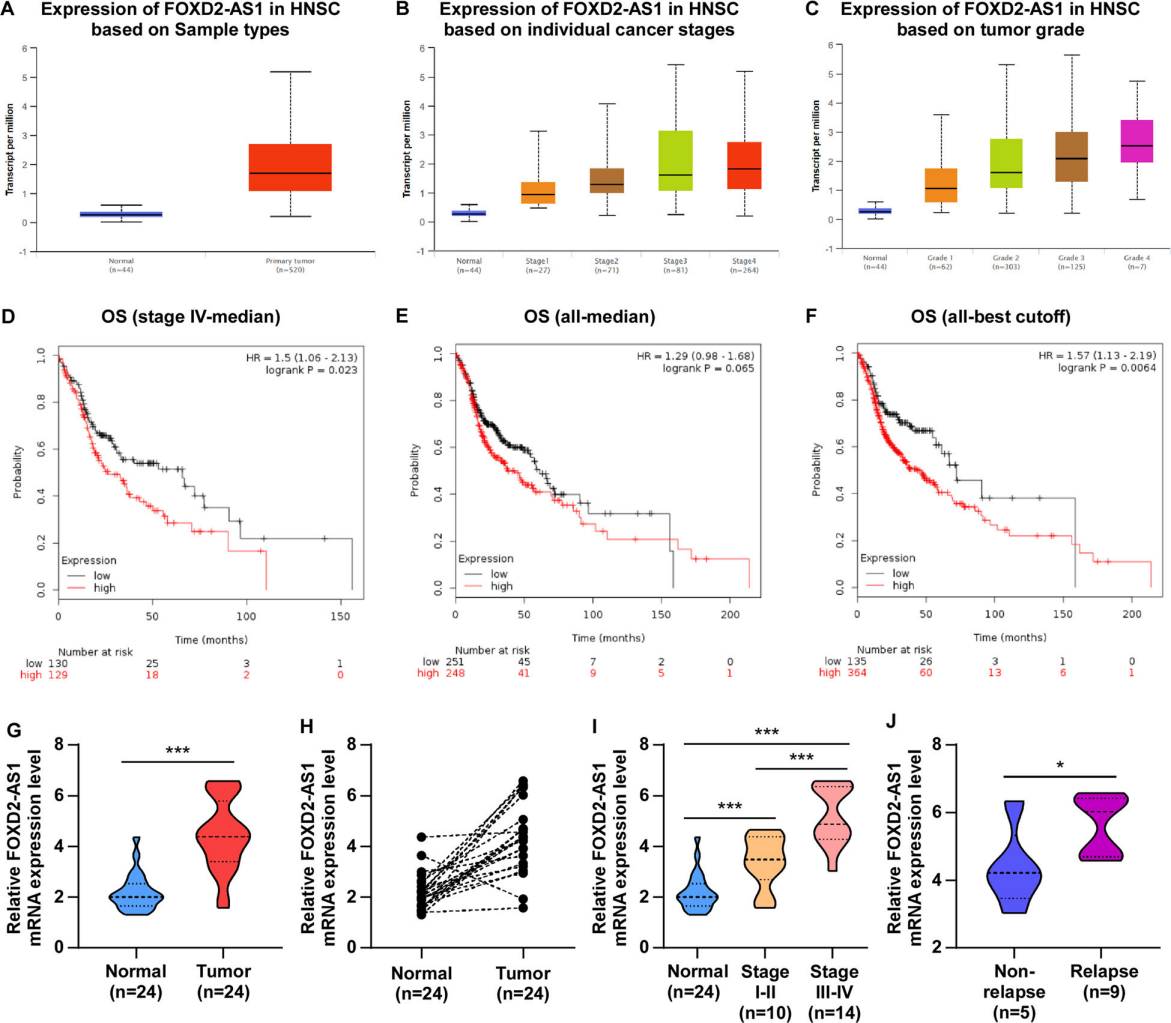
lncRNA FOXD2-AS1 were upregulated in LSCC patients with chemotherapy resistance and its high expression level was correlated with patients’ poor prognostics. **a** FOXD2-AS1 mRNA expression in primary head and neck squamous cell carcinoma (HNSC) tissues (tumor) and matched normal tissues (normal) from UALCAN webserver. **b**, **c** FOXD2-AS1 mRNA expression in primary HNSC tissues from patients with different clinical stages and tumor grades from UALCAN webserver, respectively. **d**–**f** Prognostic value of FOXD2-AS1 expression in HNSC patients by using online Kaplan–Meier Plotter analysis. **d** Overall survival (OS) of 259 stage IV HNSC patients according the FOXD2-AS1 status. The median value of FOXD2-AS1 expression levels in these 259 tissues was used to stratify the high and low expression levels of FOXD2-AS1. **e**, **f** OS of 499 HNSC patients according the FOXD2-AS1 status. The median value and the best cutoff value of FOXD2-AS1 expression levels in these 499 tissues was used to stratify the high and low expression levels of FOXD2-AS1, respectively. **g** Real-time PCR analysis of FOXD2-AS1 expression in 24 primary laryngeal squamous cancer tissues and matched adjacent normal tissues. **h** FOXD2-AS1 expression were significantly increased in 21 out of 24 cases of tumor comparing with paired normal tissues. **i** FOXD2-AS1 mRNA expression in primary HNSC tissues from patients with different clinical stages. **j** FOXD2-AS1 mRNA expression in primary HNSC tissues from patients with different relapse status. Transcript levels were normalized to GAPDH expression. *P < 0.05, **P < 0.01, and ***P < 0.001.

To define the expression pattern of lncRNA *FOXD2-AS1* in laryngeal cancer, we first analyzed the mRNA expression levels of *FOXD2-AS1* in head and neck cancer, as well as in normal tissues using a dataset from The Cancer Genome Atlas (TCGA), containing 26% of tumors from laryngeal sites, using UALCAN webserver (http://ualcan.path.uab.edu/index.html). *FOXD2-AS1* mRNA levels were significantly increased in malignant tissues compared with that in the normal tissues in the TCGA data (Fig. 1a). Moreover, the results of public data analysis indicated that increases in *FOXD2-AS1* expression levels were clearly discernible between clinical stages, with significantly higher levels in patients with more advanced stage or higher grade of head and neck cancer (Fig. 1b, c). Moreover, analysis results from Kaplan–Meier Plotter Pan-cancer RNA sequencing public webserver (http://kmplot.com/analysis/index.php?p=service&cancer=pancancer_rnaseq) indicated that higher level of *FOXD2-AS1* was significantly associated with poorer OS in all head and neck cancer cases, and stage IV head and neck cancer cases (Fig. 1d–f). We further analysis the expression pattern of FOXD2-AS1 in 24 paired of LSCC samples, comprising tumor and adjacent normal tissues form individuals, by real-time polymerase chain reaction (PCR) analysis. The results showed that FOXD2-AS1 expression was significantly upregulated in 21 out of 24 tumor tissues comparing with their adjacent normal tissues (Fig. 1g, h), and ratcheted up in later stage cancer (Fig. 1i). Since all of the 14 patients with advance laryngeal cancer (stages III and IV) were treated with cisplatin-based chemotherapy, we separated them into two groups according patients’ relapse status. An increased expression level of FOXD2-AS1 was observed in relapse group (Fig. 1j), patients in which group were considered to be resistant to chemotherapy, suggesting that FOXD2-AS1 upregulated might confer chemoresistance of LSCC patients.

Next, we verified the coding potential of *FOXD2-AS1* using a protein-coding potential assessment tool^18^. The results showed that FOXD2-AS1 does not contained a protein-coding open reading frame that is longer than 300 nt^19^, suggesting that the coding probability of FOXD2-AS1 is low (Supplementary Fig. 1a). Then, full-length *FOXD2-AS1* cDNA was cloned into pcDNA3.1 with a C-terminal Flag tag (FOXD2-AS1-Flag), according to the sequence provided by the nucleotide database (https://www.ncbi.nlm.nih.gov/nuccore/), while glyceraldehyde-3-phosphate dehydrogenase (GAPDH) with a C-terminal Flag tag (GAPDH-Flag) was used as positive control. Western blotting assay showed that Flag-tagged GAPDH could be detected in GAPDH-Flag transiently transfected 293Ft cells, whereas the FOXD2-AS1-Flag group did not show expression of a Flag-tagged protein (Supplementary Fig. 1b). Collectively, these observations confirmed that FOXD2-AS1 had no coding capability, which was consistent with previous studies^20,21^.

### FOXD2-AS1 promoted chemotherapeutic resistance of LSCC

**Fig. 2:**
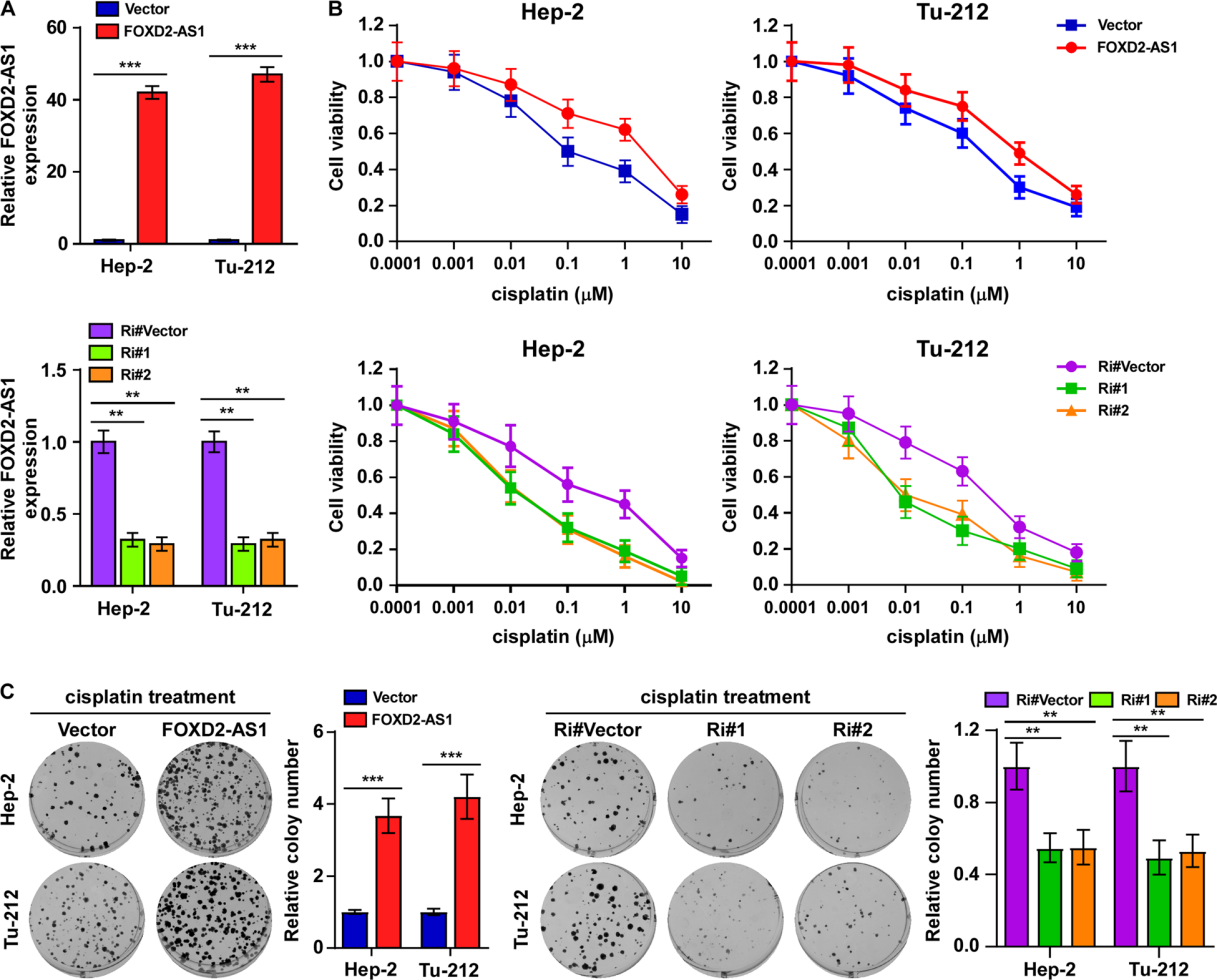
Upregulation of FOXD2-AS1 enhanced cisplatin resistance of laryngeal cancer cells in vitro. **a** Real-time PCR analysis of FOXD2-AS1 expression in indicated FOXD2-AS1-overexpressing and -silencing cells. Expression levels were normalized to GAPDH. **b** Viability of indicated cells after 24 h treatment with 0.0001–10 μM cisplatin injection. A significant decrease in viability was observed in FOXD2-AS1-silencing cells. Contrarily, cell viability was increased in FOXD2-AS1-overexpressing cells. **c** Colony formation of indicated cells with cisplatin treatment. Colony number of FOXD2-AS1-overexpressing cells was larger comparing with control cells, while colony number of FOXD2-AS1-silencing cells was strongly decreased. *P < 0.05, **P < 0.01, and ***P < 0.001.

**Fig. 3:**
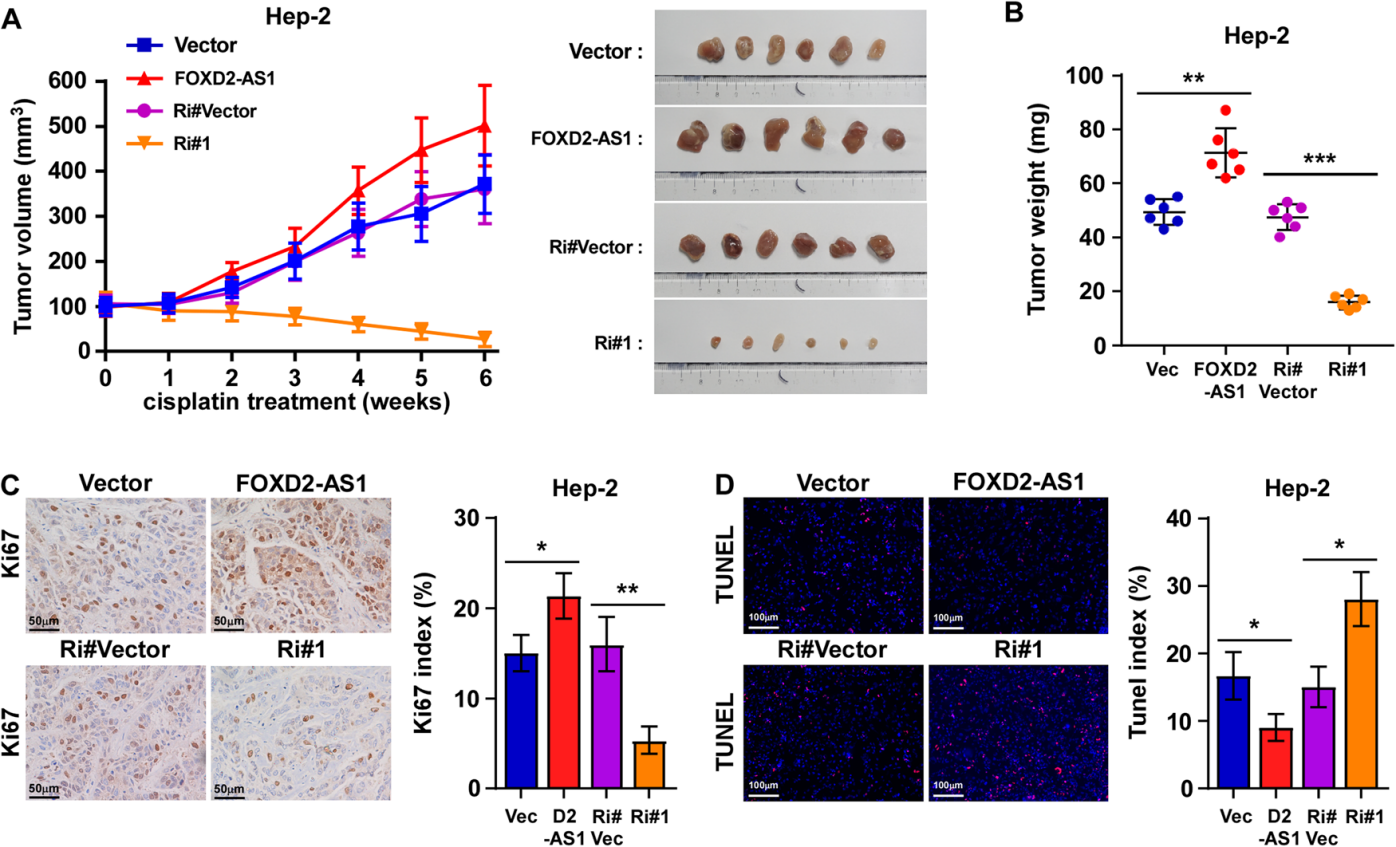
Upregulation of FOXD2-AS1 reduced sensitivity of laryngeal cancer cells to cisplatin therapy in vivo. **a**, **b** The effect of FOXD2-AS1 expression level on tumor formation during cisplatin treatment. Tumor growth curves **a** of mice injected with indicated cells were established after treated with cisplatin since 14 dpi. Mice planted with vector cells serve as controls. Subcutaneous tumors were collected **a** and the tumor weights **b** were recorded at the end point of this study. As showed in the figures, FOXD2-AS1-overexpressing cells had stronger tumor formation potential than control cells during cisplatin treatment. Representative IHC staining **c** of Ki67 and TUNEL staining **d** in paraffin-embedded specimens from the subcutaneous tumors showed significant increased Ki67 level and decreased apoptosis level in FOXD2-AS1-overexpressing cells; *n* = 6/group; dpi days post inoculation. *P < 0.05, **P < 0.01, and ***P < 0.001.

To determine the role of *FOXD2-AS1* in promoting drug resistance in LSCC, we stably expressed or knocked down *FOXD2-AS1* in laryngeal cancer cell lines to perform gain- and loss-of-function experiments in these cells, respectively. A quantitative PCR assay was used to confirm the efficiencies of overexpression and interference for *FOXD2-AS1* in Hep-2 and Tu-212 cells (Fig. 2a). Next, 3-(4,5-dimethylthiazol-2-yl)-2,5-diphenyltetrazolium bromide and colony formation assays were used to explore the sensitivity to cisplatin of the two laryngeal cancer cell lines. The results showed that *FOXD2-AS1*-overexpressing Hep-2 and Tu-212 cells were less sensitive to cisplatin (Fig. 2b, c). In contrast, knockdown of *FOXD2-AS1* increased the cells’ sensitivity to cisplatin (Fig. 2b, c). We then subcutaneously inoculated BALB/c nude mice with control Hep-2 cells, Hep-2 cells that overexpressed *FOXD2-AS1* and cells expressing FOXD2-AS1-targeting shRNA#1, respectively. When the tumor volume reached roughly 100 mm^3^, mice were administrated with cisplatin (5 mg/kg). The results showed that increased *FOXD2-AS1* facilitated the growth of Hep-2 cells in response to chemotherapy (Fig. 3a–c). By contrast, *FOXD2-AS1* silencing using shRNAs rescued the chemotherapeutic sensitivity of the Hep-2 cells, resulting decreased tumor volumes and better survival of the mice (Fig. 3a–c). Moreover, immunohistochemistry (IHC) analysis and terminal deoxynulceotidyl transferase nick-end-labeling (TUNEL) staining of harvested xenografts revealed that upregulation of *FOXD2-AS1* increased laryngeal cancer cell proliferation, and significantly decreased cell apoptosis level in LSCC (Fig. 3d, e). Thus, these findings indicated that upregulation of *FOXD2-AS1* enhanced chemotherapy resistance in LSCC.

### FOXD2-AS1 was essential for stem cell-like properties of LSCC cells

**Fig. 4:**
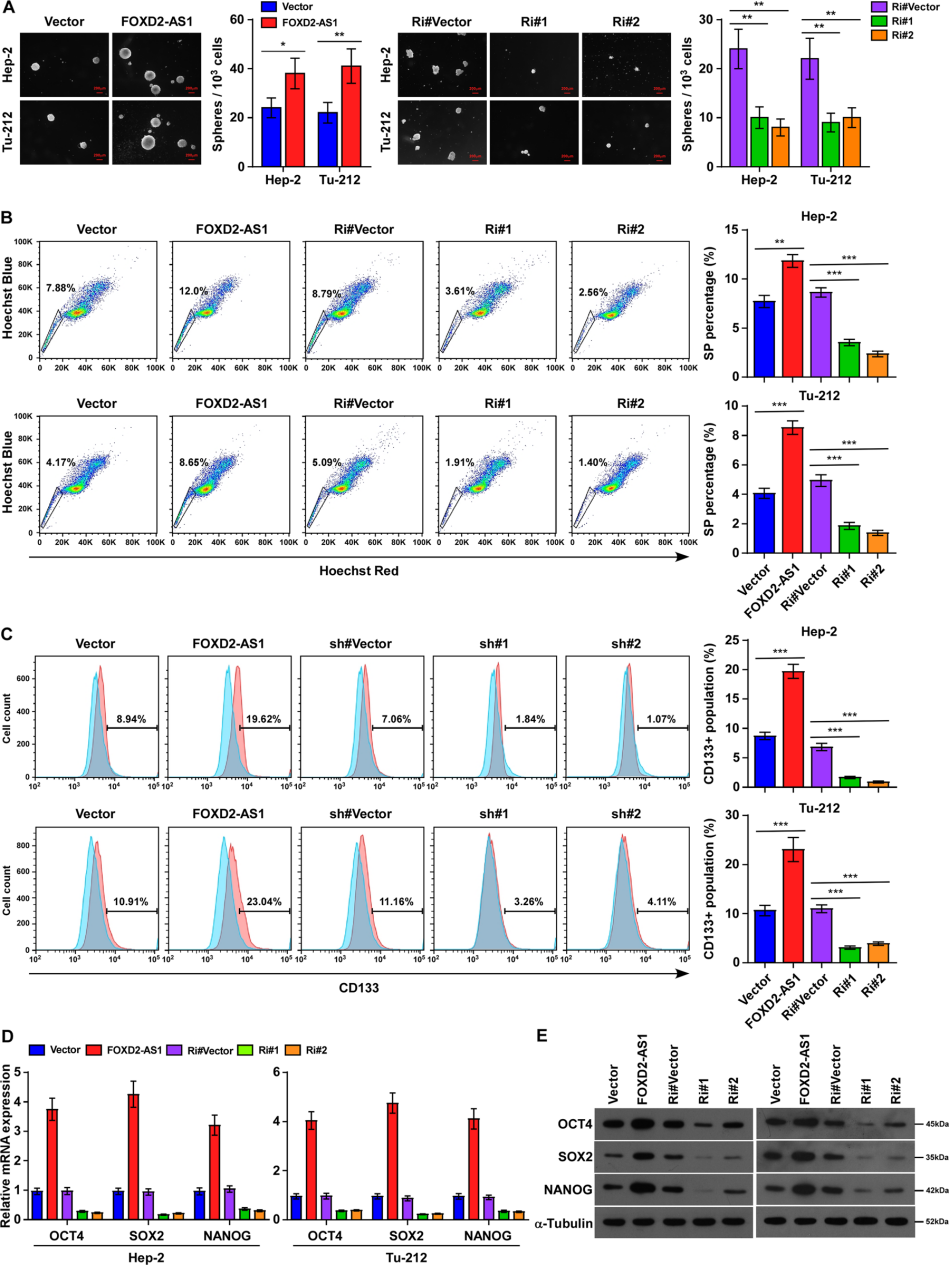
Upregulation of FOXD2-AS1 increased CSC-like potential in laryngeal cancer. **a** Representative micrographs and quantification of tumor spheres formed by indicated Hep-2 and Tu-212 cells. Histograms showed the mean number of spheres formed by the indicated cells. **b**, **c** Flow cytometry analyses showed proportion of side population **b** and CD133^+^ population **d** in laryngeal cancer cells. **d** Real-time PCR analysis of OCT4, SOX2, and NANOG expression in FOXD2-AS1-overexpressing and -silencing cells. Expression levels were normalized to GAPDH. **e** Western blot analysis of OCT4, SOX2, and NANOG expression in indicated cells. GAPDH was used as a loading control. *P < 0.05, **P < 0.01, and ***P < 0.001.

Cancer stemness, which is determined by the proportion of CSCs in tumors, is responsible for the development of resistance to chemotherapy. To further reveal the relationship between lncRNA *FOXD2-AS1* and LSCC progression, we performed several experiments to assess the stem cell-like properties of LSCC cells. Intriguingly, we found that upregulation of *FOXD2-AS1* dramatically increased the size and number of spheres among Hep-2 cells, while knockdown of *FOXD2-AS1* suppressed sphere formation (Fig. 4a). Increased side population (SP) efflux properties are linked to tumor stem-like cells; therefore, we applied a flow cytometry SP discrimination assay on laryngeal cancer cells to visualize SP cells as a dim tail of events with decreased fluorescence in two Hoechst channels^22^. As shown in Fig. 4b, flow cytometry analyses revealed the presence of SP cells in parental Hep-2 and Tu-212 cells, and in *FOXD2-AS1*-overexpressing Hep-2 and Tu-212 cells. The percentage of SP cells in *FOXD2-AS1*-overexpressing Hep-2 and Tu-212 cells was larger than that in the parental cells. CD133 expression has been demonstrated as a marker of CSC-like populations in many tumors, including laryngeal cancer; therefore, the effect of *FOXD2-AS1* on the CD133-positive cell population of LSCC was detected using immunofluorescence. The analysis showed that *FOXD2-AS1* overexpression induced the CD133-positive cell population in LSCC cells, while *FOXD2-AS1* downregulation showed the opposite effect (Fig. 4c). Consistently, octamer-binding protein 4 (OCT4), SRY-box 2 (SOX2), and nanog homeobox (NANOG), which are transcription factors that are upregulated in various types of cancer and are involved in cancer stemness maintenance, were upregulated at both the mRNA and protein levels in *FOXD2-AS1*-overexpressing LSCC cells, but were downregulated in *FOXD2-AS1*-silenced cells, compared with those in the control (Fig. 4d, e). These results suggested that *FOXD2-AS1* is required for the maintenance of the stem cell-like properties of LSCC cells.

### FOXD2-AS1 bound to STAT3 and augmented its transcriptional activity

**Fig. 5:**
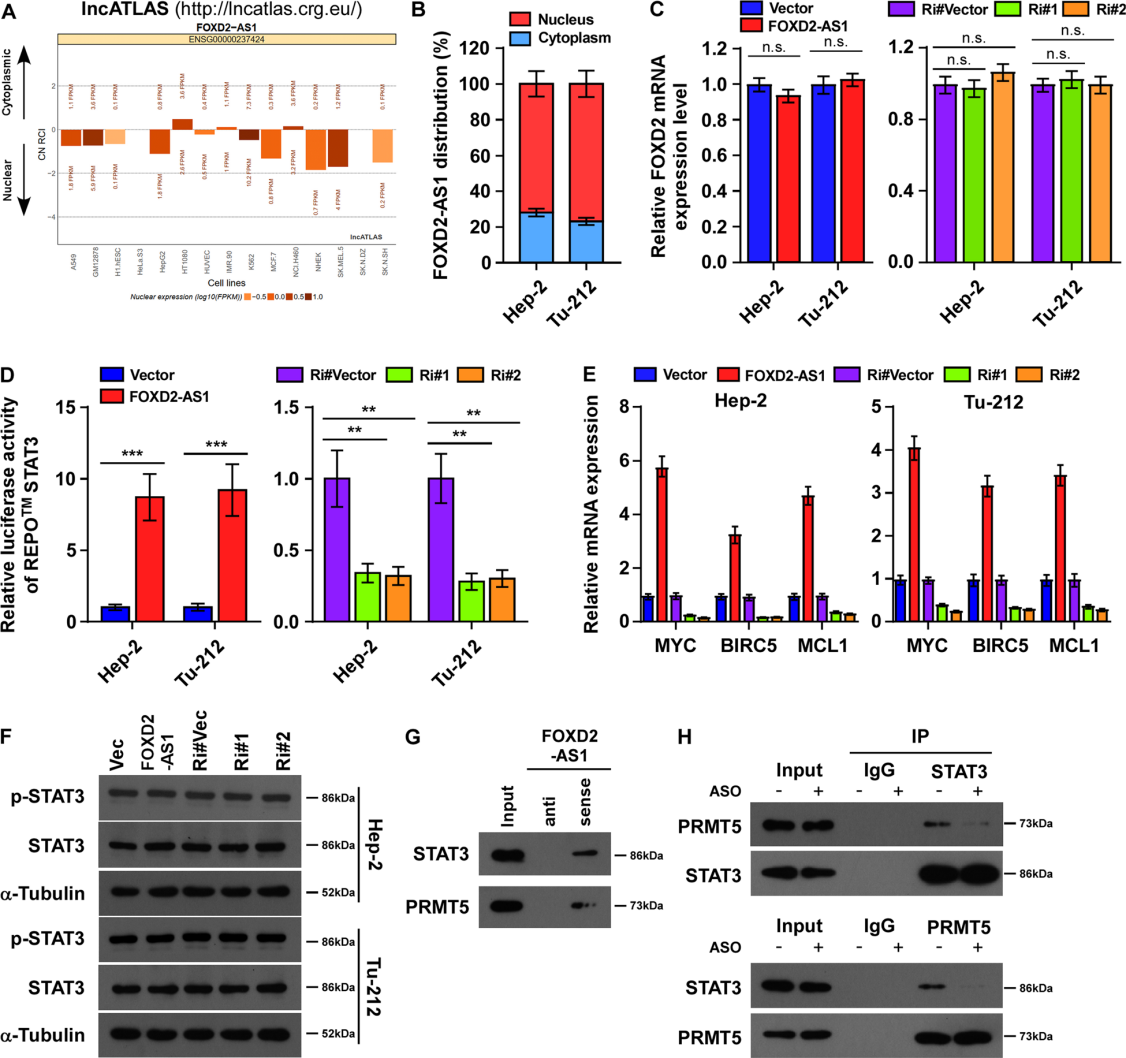
Upregulation of FOXD2-AS1 bound to STAT3 and augmented STAT3 transcriptional activity by recruiting PRMT5. **a** Distribution of FOXD2-AS1 was predicted by public webserver lncATLAS. **b** The expression levels of FOXD2-AS1 were determined by qRT-PCR in cytoplasmic and nuclear fractions of laryngeal cancer cells. The percentages of FOXD2-AS1 distribution in cytoplasm and nucleus were shown. **c** Real-time PCR analysis of FOXD2 was performed, showing that FOXD2-AS1 would not affect the expression of its host gene. **d** REPO^TM^ STAT3 luciferase assay to assess STAT3 activation in the indicated cells transfected with STAT3 luciferase reporter plasmid, showing an increased activity of STAT3 in FOXD2-AS1-overexpressing cells. **e** Real-time PCR analysis of STAT3 target genes (MYC, BIRC5, and MCL1) mRNA expression in FOXD2-AS1-overexpressing and -silencing cells. Expression levels were normalized to GAPDH. **f** Western blot analysis of STAT3, p-STAT3 expression in indicated cells. Alpha-tubulin was used as a loading control. **g**, **h** RNA pull-down assay and co-immunoprecipitation (Co-IP) assay were performed to assess the relationship between FOXD2-AS1, STAT3, and PRMT5. **g** SDS–PAGE analysis of eluted proteins after RNA pull-down, showing that both STAT3 and PRMT5 were presented in the FOXD2-AS1 pull-down fraction. **h** Co-IP of STAT3 and PRMT5 was performed in FOXD2-AS1-silencing and control cells, showing that FOXD2-AS1 depletion disrupted the combination of STAT3 and PRMT5. *P < 0.05, **P < 0.01, and ***P < 0.001.

Next, we investigated the precise mechanism of *FOXD2-AS1* in regulating cancer stemness. The subcellular localization of the lncRNA could provide insights into its functions; therefore, we browsed the annotations of *FOXD2-AS1* at the LncATLAS webserver (http://lncatlas.crg.eu/). The online data indicated that *FOXD2-AS1* is primarily located in the nucleus of several types of cells (Fig. 5a). We then detected the cellular distribution of *FOXD2-AS1* in Hep-2 and Tu-212 cells by performing quantitative real-time reverse transcription-PCR (qRT-PCR) analysis in cytoplasmic and nuclear fractions of cells. The percentages of *FOXD2-AS1* distribution in cytoplasm and nucleus were shown in Fig. 5b, which revealed that *FOXD2-AS1* is expressed and distributed predominantly in the nucleus of these cells. Next, the mRNA expression of *FOXD2* (encoding forkhead box D2), which is the neighboring protein-coding gene to *FOXD2-AS*1, was verified in *FOXD2-AS1*-overexpressing or -silenced cells. Results showed that the *FOXD2* mRNA levels did not change in response to alterations in *FOXD2-AS1* expression (Fig. 5c), suggesting FOXD2-AS1 would not act as a cis-regulator in laryngeal cancer. Recent studies have reported that nuclear lncRNAs can execute their cellular functions by acting as an RNA scaffold^23^. We hypothesized that nuclear *FOXD2-AS1* may bind to cancer stemness regulators directly to either stabilize or activate them.

Previous studies indicated that STAT3 plays an important role in the EMT and self-renewal of laryngeal CSCs, acting as a mediator that transduce intracellular and extracellular signals to the nucleus^24–26^. Interestingly, we found that upregulation of *FOXD2-AS1* significantly induced STAT3 reporter activity, while it had no effect on TCF/LEF or Notch reporter activity, which reflect the activation level of Wnt signaling and Notch signaling, two important signaling pathways in cancer stemness regulation (Fig. 5d, Supplementary Fig. 2a, b). Consistently, the mRNA expression levels of STAT3 downstream target genes MYC, BIRC5, and MCL1 were significantly increased in FOXD2-AS1-overexpressing cells (Fig. 5e). Therefore, we sought to clarify the relationship between *FOXD2-AS1* and STAT3 signaling. Intriguingly, the results of western blotting shown that the levels of phosphorylated STAT3 (p-STAT3) and total STAT3 were not change by *FOXD2-AS1* in Hep-2 cells (Fig. 5f), and the mRNA levels of *STAT3* were consistent in *FOXD2-AS1*-overexpressing Hep-2 and vector cells (Supplementary Fig. 2c). We then performed RNA pull-down and western blotting assay to verify the RNA–protein interaction between *FOXD2-AS1* and STAT3. As shown in Fig. 5g, STAT3, along with protein arginine methyltransferase-5 (PRMT5), a key transcriptional cofactor of STAT3 that can improve its transcriptional activity to downstream targeted genes, appeared in *FOXD2-AS1* precipitates, suggesting that FOXD2-AS1, STAT3, and PRMT5 were consistent with the notion of a ternary complex. More importantly, *FOXD2-AS1* knockdown remarkably disrupted the combination of STAT3 and PRMT5 (Fig. 5h), and reduced transcriptional activity of STAT3 and diminished the expression of downstream CSC markers (Fig. 4d). Thus, *FOXD2-AS1* is important for STAT3 activation and is also required for interaction between STAT3 and PRMT5.

### FOXD2-AS1 enhanced cancer stemness and chemoresistance of LSCC through STAT3

**Fig. 6:**
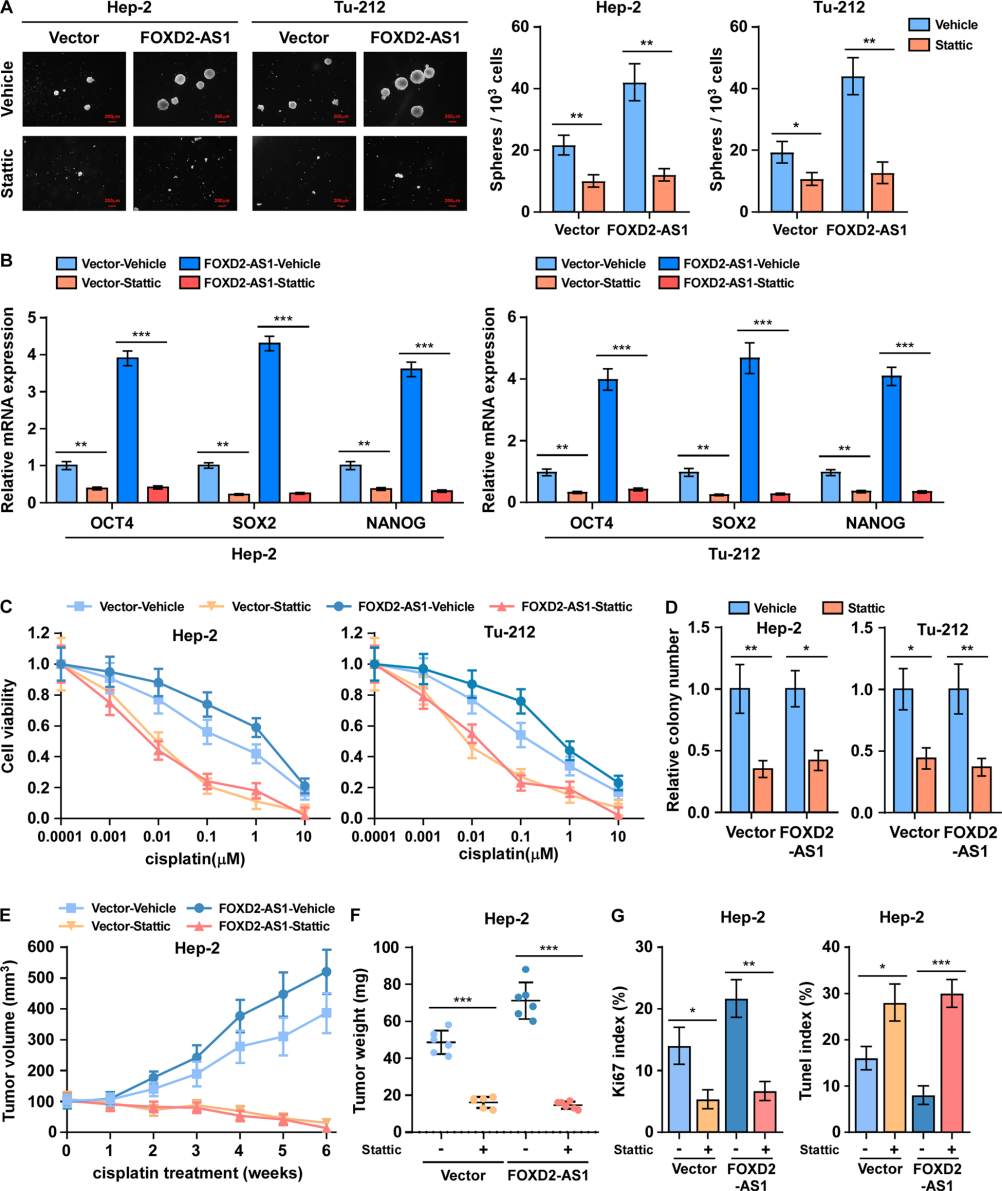
FOXD2-AS1 conferred cancer stemness and chemotherapy resistance in laryngeal cancer through STAT3 activation. **a** Representative micrographs and quantification of tumor spheres formed by FOXD2-AS1-overexpressing laryngeal cancer cells treated with vehicle or STAT3 inhibitor stattic. Histograms showed the mean number of spheres formed by the indicated cells. **b** Real-time PCR analysis of OCT4, SOX2, and NANOG expression in indicated cells that under stattic treatment and control cells. Expression levels were normalized to GAPDH. **c** Viability of indicated cells after 24 h cisplatin treatment or treating with cisplatin plus stattic, implicating that stattic-abolished FOXD2-AS1 induced cisplatin resistance of laryngeal cancer cells. **d** Colony formation of FOXD2-AS1-overexpressing cells with indicated treatment. **e**, **f** The effect of STAT3 inhibition by stattic on tumor formation of FOXD2-AS1-overexpressing laryngeal cancer cells during cisplatin treatment (*n* = 6/group). Stattic treatment significantly rescued the sensitivity to cisplatin in FOXD2-AS1-overexpressing cells. **g** IHC staining of Ki67 and TUNEL staining were performed to verified the proliferation and anti-apoptosis potential of FOXD2-AS1-overexpressing cells accompanied with indicated treatment. *P < 0.05, **P < 0.01, and ***P < 0.001.

Next, we disrupted the STAT3 pathway to verify whether STAT3 is the most important effector of *FOXD2-AS1* in cancer stemness and chemoresistance regulation. As anticipated, treatment with stattic, a small-molecule inhibitor of STAT3 activation and dimerization, abrogated the effects of *FOXD2-AS1* in CSC-like property promotion. The sphere formation potentials of LSCC cells were impaired by stattic, even in the context of *FOXD2-AS1* overexpression (Fig. 6a). The expression levels of cancer stemness markers, NANOG, OCT4, and SOX2, were significantly downregulated by stattic in *FOXD2-AS1*-overexpressing cells and vector cells (Fig. 6b). Moreover, stattic treatment enhanced the chemotherapeutic toxicity of cisplatin to LSCC cells in vitro (Fig. 6c, d) and in vivo (Fig. 6e–g, Supplementary Fig. 3a), which could not be rescued by upregulating *FOXD2-AS1*. Taken together, the results showed that *FOXD2-AS1* was involved in cancer stemness maintenance and chemoresistance promotion in a manner that is strongly dependent on STAT3.

## Discussion

In the present study, we identified a novel lncRNA, *FOXD2-AS1*, as the important mediator of cancer stemness maintenance and chemoresistance promotion in LSCC. A higher expression of *FOXD2-AS1* correlated positively with a worse response to chemotherapy by patents with LSCC. Further mechanistic study revealed that upregulated *FOXD2-AS1* acted as a scaffold for STAT3 and PRMT5, resulting in increased transcriptional activity of STAT3, which contributed to stem cell-like properties of LSCC cells. Furthermore, we showed that inhibiting *FOXD2-AS1* using targeted shRNAs in LSCC cells could recover the chemotherapy sensitivity of tumor cells. Therefore, our results suggested the importance of lncRNA *FOXD2-AS1* as a novel therapeutic target in LSCC.

Increasing evidence shows that lncRNAs are crucial in diverse pathological contexts, rather than being the “junk” products of pre-mRNA splicing^27,28^. Many lncRNAs have been reported to exert a regulatory role in tumor progression. Importantly, dysregulated lncRNAs have also been linked to therapeutic resistance of breast cancer^29^, lung cancer^30^, glioma^31^, esophageal cancer^32^, and other carcinomas^33^. Our results clearly demonstrated that upregulation of *FOXD2-AS1* was a frequent oncogenic event in LSCC, contributing to the reduced sensitivity of LSCC to chemotherapy. Furthermore, gain- and loss-of-function studies showed that upregulated *FOXD2-AS1* maintained cancer stemness in LSCC and promoted the cells’ resistance to cisplatin treatment. Therefore, our clinical and functional studies strongly supported an oncogenic role of *FOXD2-AS1* in chemotherapy resistance.

Recently, the characteristic molecular functions of lncRNAs have been identified, including acting as signals, decoys, guides, and scaffolds^23,34^. These archetypal functions of lncRNAs could represent a useful framework to consider how lncRNAs acquire properties as biological signal transducers, which are determined by their subcellular locations. However, only a few lncRNAs have been reported to play roles in LSCC^9,35–38^ and most of these studies lacked convincing experimental support for the underlying mechanisms^39^. To better understand the regulatory mechanisms of *FOXD2-AS1* in LSCC chemotherapy resistance, we showed that: (i) *FOXD2-AS1* is predominantly located in the nuclei of LSCC cells; (ii) *FOXD2-AS1* does not act as a cis-regulator of neighbor genes; (iii) *FOXD2-AS1* can bind to with STAT3; and (iv) *FOXD2-AS1* is required for the interaction between STAT3 and PRMT5, and is involved in regulating the transcriptional activity of STAT3. According to these experimental data, we proposed that *FOXD2-AS1* functions as a scaffold for certain proteins, such as STAT3, and contributes to signal transduction in LSCC.

The excessive activation of STAT3 signaling has been observed in many types of cancer, and is considered as one of the most frequent impact factors in cancer stemness maintenance^40–43^. When the STAT3 signaling pathway is activated, STAT3 is phosphorylated by receptor-associated kinases and subsequently translocates to the cell nucleus where it activates the transcription of target genes that regulate cell stemness^44–46^. Although multiple mediators, including interleukin-6^46^, G-protein-coupled receptors^47^, Toll-like receptors^48^, and microRNAs^49^, were identified to regulate STAT3 signaling in cancer, we have a limited understanding of the upstream regulatory mechanisms of STAT3 in LSCC. The results of the present study revealed that *FOXD2-AS1*-promoted cancer stemness of LSCC cells relies on STAT3 transcriptional activation. The positive correlation between the expression level of *FOXD2-AS1* and STAT3 signaling activity supported the hypothesis that the *FOXD2-AS1*-STAT3 axis plays a critical regulatory role in the maintenance of the CSC proportion in LSCC. Thus, we demonstrated that nuclear-localized *FOXD2-AS1* directly bound to STAT3 in LSCC cells, which activated the STAT3 signaling pathway, and upregulated the levels of stemness-associated factors, including NANOG, SOX2, and OCT4. Moreover, previous studies showed that STAT3 could regulate gene expression through epigenetic mechanisms^50,51^. Our results showed that *FOXD2-AS1* served as scaffold to maintain the binding of STAT3 to PRMT5, an epigenetic regulator that is essential for transcriptional regulation by promoting the methylation of the guanidino nitrogen of arginyl residues present in histones. Collectively, these data suggested that *FOXD2-AS1* is a regulator of STAT3 that functions to control the maintenance of cancer stemness and chemotherapy resistance in LSCC.

**Fig. 7:**
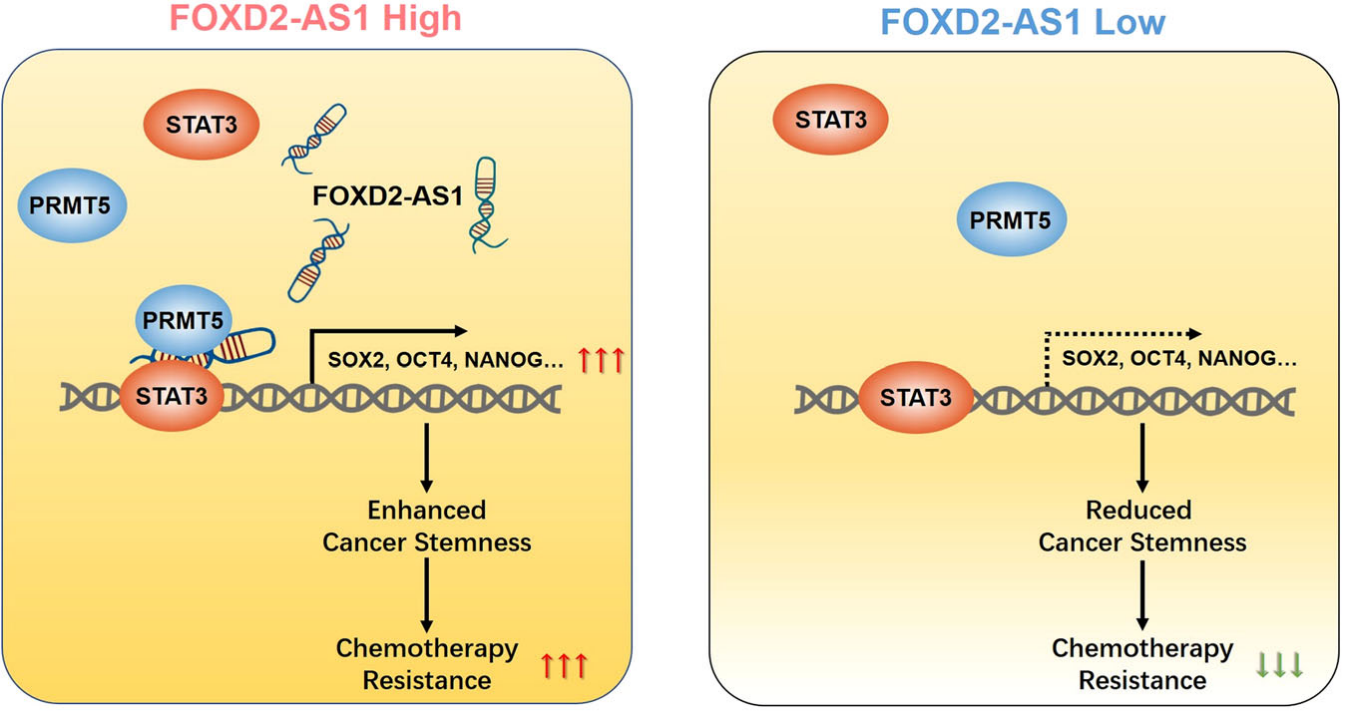
Proposed mechanism of FOXD2-AS1-mediated STAT3 activation in LSCC. Upregulated FOXD2-AS1 promoted transcriptional activity of STAT3, by serving as scaffold of STAT3 and PRMT5, and subsequently conferred the cancer stemness and chemoresistance in LSCC.

In summary, this study provided compelling evidence that lncRNA *FOXD2-AS1* is a mediator of LSCC chemotherapeutic resistance promotion. *FOXD2-AS1* functions by activating the STAT3 signaling pathway, thus increasing the expression of stemness-associated factors to maintain the CSC proportion in LSCC, which is illustrated in Fig. 7. Our findings offer a novel therapeutic target, *FOXD2-AS1*, to improve the current treatment status of human LSCC.

## Materials and methods

### Cell culture

Human LSCC cell line Hep2 was obtained from CoBioer (Nanjing), and the TU-212 cell line was obtained from XINYU BIOLOGICAL TECHNOLOGY CO., LTD. (Shanghai). Both of the two cells were cultured in Dulbecco’s Modified Eagle Medium (DMEM) supplemented with 10% fetal bovine serum (FBS; Hyclone, South Logan, UT) and maintained in an incubator at 37 °C with 5% CO_2_ and 100% humidity with the medium changed every other day.

### Patients and tissue specimens

Twenty-four cases of fresh laryngeal squamous cancer tissues and adjacent normal tissues were collected for this study, including nine chemotherapeutic resistant tissues from patients that had relapse after receiving chemotherapy, and five chemotherapeutic sensitive tissues from non-relapse patients, which were frozen and stored in liquid nitrogen until required. No samples were excluded in this study. Prior patients’ consents and approval from the Institutional Research Ethics Committee were obtained for the use of these clinical specimens in research.

### RNA purification and real‐time quantitative PCR validation

Total RNA was extracted from cultured cells or tissues using the TRIzol reagent (Invitrogen, Carlsbad, CA) according to the manufacturer’s instructions. One microgram of extracted RNA from each sample was used for cDNA synthesis with HiScript III 1st Strand cDNA Synthesis Kit (Vazyme, Nanjing, China). cDNAs were amplified and quantified by TB Green® *Premix Ex Taq*™ II (Takara, Japan) in CFX96 Real Time System C1000 Cycler (Bio‐Rad Laboratories, Singapore). Expression data were normalized to the housekeeping gene GAPDH and calculated as 2^−[(Ct of gene) − (Ct of GAPDH)]^, where Ct represents the threshold cycle for each transcript. Primer sequences were listed in the Supplementary Table 1. All experiments were carried out at least twice with three or more technical triplicates for each.

### Plasmids, virus constructs, and retroviral infection

Human FOXD2-AS1 (NR_026878.1), FOXD2-AS1-Flag, GAPDH (NM_002046.7), and GAPDH-Flag cDNAs were PCR amplified and cloned into the pMSCV‐puro‐retro vector (Clontech, Beijing, China). Two shRNAs against FOXD2-AS1 in pLKO.1‐puro vector were purchased from Transheep Bio (Shanghai, China). pMSCV-IRES-GFP II (pMIG II) was a gift from Dario Vignali (Addgene plasmid #52107). Transfections of above plasmids were performed by the Lipofectamine 3000 reagent (Invitrogen, Carlsbad, CA) according to the manufacturer’s instructions. A total of 2 × 10^5^ cells were seeded and infected by retrovirus generated by pMSCV‐puro‐cDNAs or lentivirus generated by pLKO.1‐puro‐shRNAs for 72 h. The stable cell lines were selected with 0.5 μg/mL puromycin and 250 μg/mL G418 for 7 days.

### Western blot

Cells or tissues were harvested in sampling buffer [62.5 mmol/L Tris-HCl (pH 6.8), 10% glycerol, 2% and sodium dodecyl sulfate (SDS)]. Protein concentration was determined with the bicinchoninic acid Protein Assay Kit (Pierce, USA) according to the manufacturer’s instructions. Equal quantities of protein were separated electrophoretically on 10% SDS/polyacrylamide gels (PAGs) and transferred onto polyvinylidene difluoride (PVDF) membranes (Roche). The PVDF membrane was hybridized with the following primary antibodies overnight at 4 °C: anti‐OCT4 (1:1000, #75463, CST), anti‐NANOG (1:2000, #4903, CST), anti‐SOX2 (1:1000, #4900, CST), anti‐GAPDH (1:1000, #5174, CST), anti‐STAT3 (1:1000, #9139, CST), anti‐phosphorylated-STAT3 (1:1000, #9145, CST), and anti‐PRMT5 (1:1000, #79998, CST). Secondary antibodies: goat anti‐rabbit immunoglobulin G (1:2000, Pierce, USA), and goat anti‐mouse immunoglobulin G (1:2000, Pierce, USA) were used in this study. Protein expression was detected by enhanced chemiluminescence (Pierce) according to the manufacturer’s suggested protocols. All experiments were carried out at least twice with three or more technical triplicates for each.

### Sphere formation and colony formation assays

For examining spheroid formation potentials, a total of 1000 cells were suspended in sphere culture medium (SCM) (containing DMEM/F12 serum-free medium(Invitrogen), 2% B-27 Supplement (Invitrogen), 20 ng/ml basal fibroblast growth factor (bFGF) (PeproTech, Rocky Hill, NJ, USA), 20 ng/ml epidermal growth factor (EGF) (PeproTech), 0.4% bovine serum albumin (BSA) (Sigma-Aldrich), and 5 mg/ml insulin (SigmaAldrich)) for 10 days and the number of spheroids larger than 50 μm were counted. For colony formation assay, cells were planted in six-well plates (in triplicate at 200 cells per well), and cultured with complete medium for 10 days. After most of the colonies had expanded to >50 cells, they were washed twice with phosphate-buffered saline (PBS), fixed in methanol for 15 min, and dyed with crystal violet for 15 min at room temperature. After washing out the dye and drying, the plates were photographed. The software Image J was used to quantify the colonies objectively.

### SP discrimination assay

Cells were analyzed by FACS during logarithmic growth phase (24 h after replanting). Cells were digested with 0.25% trypsin (Sigma-Aldrich), washed twice with calcium/magnesium-free PBS, resuspended in fresh DMEM (supplemented with 2% FBS) at a concentration of 1 × 10^6^ cells/mL. The DNA binding dye, Hoechst 33342 (Sigma-Aldrich, St. Louis, MO), was then added at a final concentration of 5 µg/mL and the samples were incubated in the dark for 45 min with periodic mixing. The cells were then washed twice with ice-cold PBS, and kept at 4 °C in dark prior to sorting by a Moflo XDP (Beckman Coulter, Fullerton, CA). Because Hoechst 33342 extrudes from cells treated with verapamil (a calcium ion tunnel antagonist)-sensitive ABC transporters, a subset of the cells were incubated with 50 µmol/L verapamil for 30 min at 37 °C before the addition of Hoechst 33342 to determine whether this would block the fluorescent efflux of SP cells in the samples.

### Immunoprecipitation

A total of 1 × 10^6^ cells were harvested by 1 mL lysis buffer (150 mM NaCl, 1% NP‐40, 1% sodium deoxycholate, 0.1% SDS, and 50 mM Tris–HCl [pH = 7.5]) in the presence of protease inhibitor (Roche) and phosphatase inhibitor (Sigma). The anti-STAT3 antibody (2.5 μg/reaction), anti-PRMT5 antibody (2.5 μg/reaction), and corresponding IgG control (2.5 μg/reaction, sc‐2025, Santa Cruz Biotechnology) was added to lysates and samples were incubated at 4 °C overnight before adding 40 μL Protein‐G Dynabeads (Invitrogen) for 1 h at 4 °C. The IP samples were washed by wash buffer (20 mM Tris–HCl (pH = 7.5), 150 mM NaCl 0.1% Triton‐x100, and 10% glycerol) three times before electrophoresis.

### Tumor formation in an animal model

The animal studies were approved by the Ethics Committee, and all the experiments conform to the relevant regulatory standards. Nude mice were purchased from the Shanghai Slac Laboratory Animal Co. Ltd and maintained in microisolator cages. All animals were used in accordance with institutional guidelines and the current experiments were approved by the Use Committee for Animal Care. In the tumor model, male BALB/c nude mice (5-week old, 15–18 g) were randomly divided into four groups (*n* = 6 mice/group). The indicated cells (Hep-2/Vector cells [1 × 10^6^], Hep-2/FOXD2-AS1 cells [1 × 10^6^], Hep-2/Ri #Vector cells [1 × 10^6^], or Hep-2/Ri #1 cells [1 × 10^6^]) were implanted subcutaneously into the nude mice. After the volume of xenograft tumors reached ~100 mm^3^, mice were treated with intraperitoneal injection of cisplatin (10 mg/kg) three times per week (as per cycle), for up to 6 weeks. Tumor volumes were detected weekly in a blinded fashion. Upon experimental end point, animals were euthanized, tumors were excised, weighed, paraffin‐embedded, and sectioned for IHC and hematoxylin–eosin staining.

### Statistical analysis

Independent sample *t*‐tests were performed to compare continuous variables between two groups, and a *χ*^2^ test was applied for comparison of dichotomous variables. Values of *P* < 0.05 were considered significant. The data are presented as the mean ± SD or else described in figure legends. For animal studies, no statistical method was used to predetermine sample size. The experiments were not randomized.

## Supplementary information

Supplementary Figure Legends

Supplementary Figure 1

Supplementary Figure 2

Supplementary Figure 3

Supplementary Table

Supplementary

Supplementary

Supplementary
