# Supplementary material for: Long noncoding RNA FOXD2-AS1 enhances chemotherapeutic resistance of laryngeal squamous cell carcinoma via STAT3 activation
Source: Cell Death Dis. 2020 Jan 20;11(1):41. doi: 10.1038/s41419-020-2232-7 (PMC6971019; doi:10.1038/s41419-020-2232-7)
Supplement: Supplementary file 1 — Supplementary Figure Legends [file 41419_2020_2232_MOESM1_ESM.docx]

**Supplementary Figure Legends**

**Supplementary Figure 1.** (A-B) FOXD2-AS1 does not possess protein coding potential.

**Supplementary Figure 2.** (A) FOXD2-AS1 does not affect the activity of Wnt signaling pathway and Notch signaling pathway. (B) FOXD2-AS1 does not alter STAT3 transcription level.

**Supplementary Figure 3.** (A) Tumor from each mouse. Stattic treatment abolished FOXD2-AS1-induced cisplatin resistance of LSCC.
