# Supplementary figures and images for: Long noncoding RNA FOXD2-AS1 enhances chemotherapeutic resistance of laryngeal squamous cell carcinoma via STAT3 activation
Source: Cell Death Dis. 2020 Jan 20;11(1):41. doi: 10.1038/s41419-020-2232-7 (PMC6971019; doi:10.1038/s41419-020-2232-7)

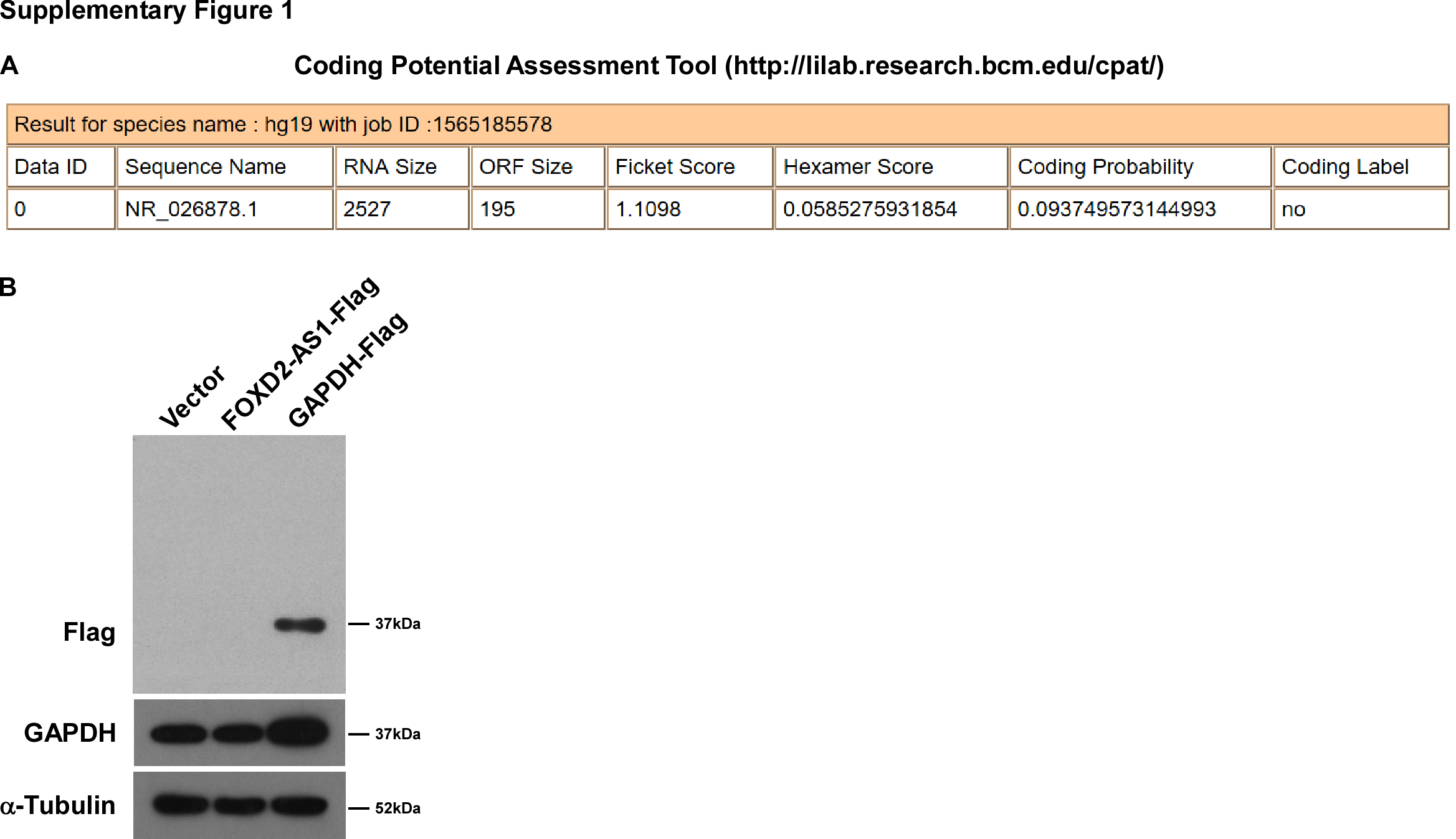

Supplement: Supplementary file 2 — Supplementary Figure 1 [file 41419_2020_2232_MOESM2_ESM.tif]

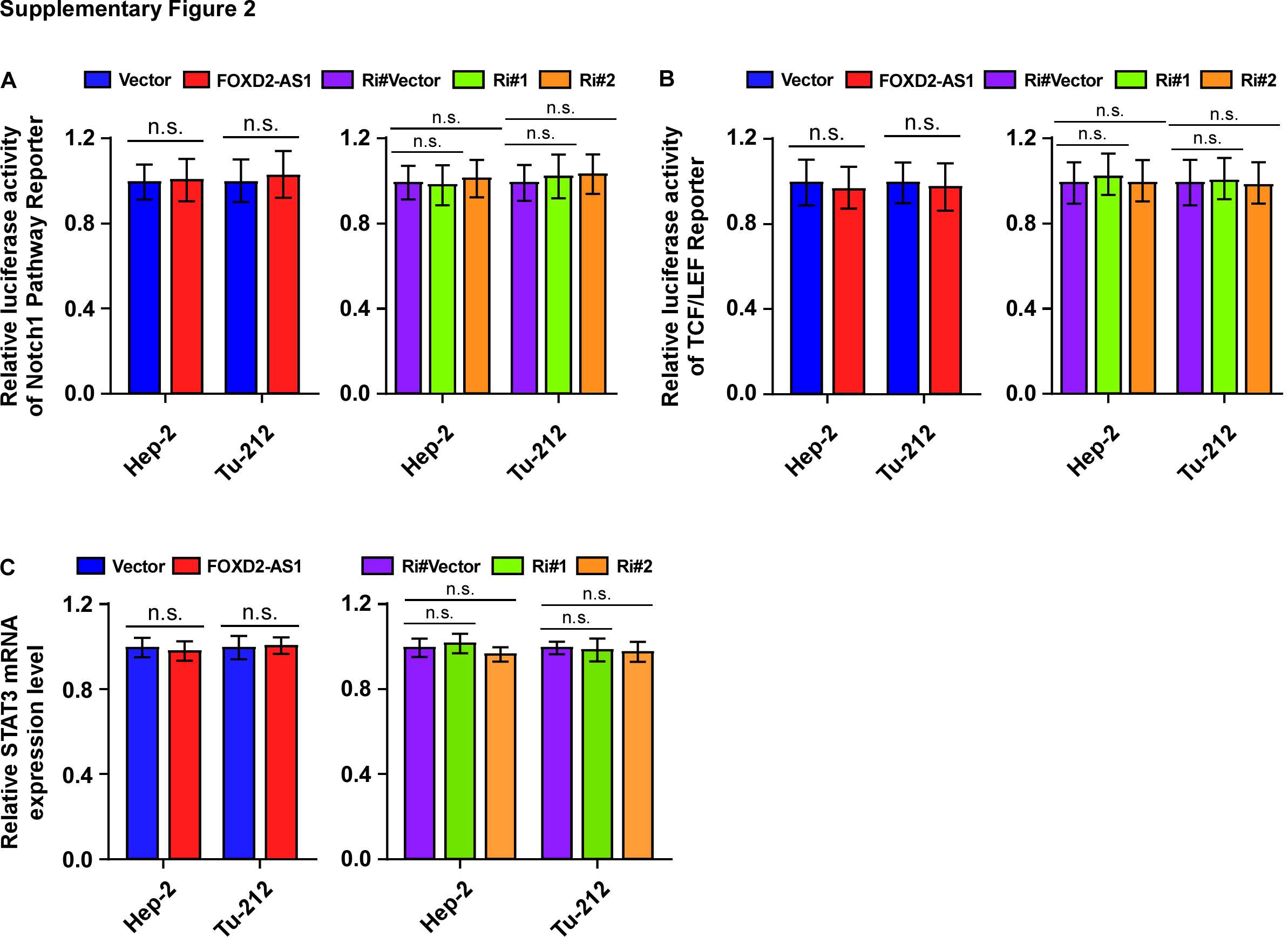

Supplement: Supplementary file 3 — Supplementary Figure 2 [file 41419_2020_2232_MOESM3_ESM.tif]

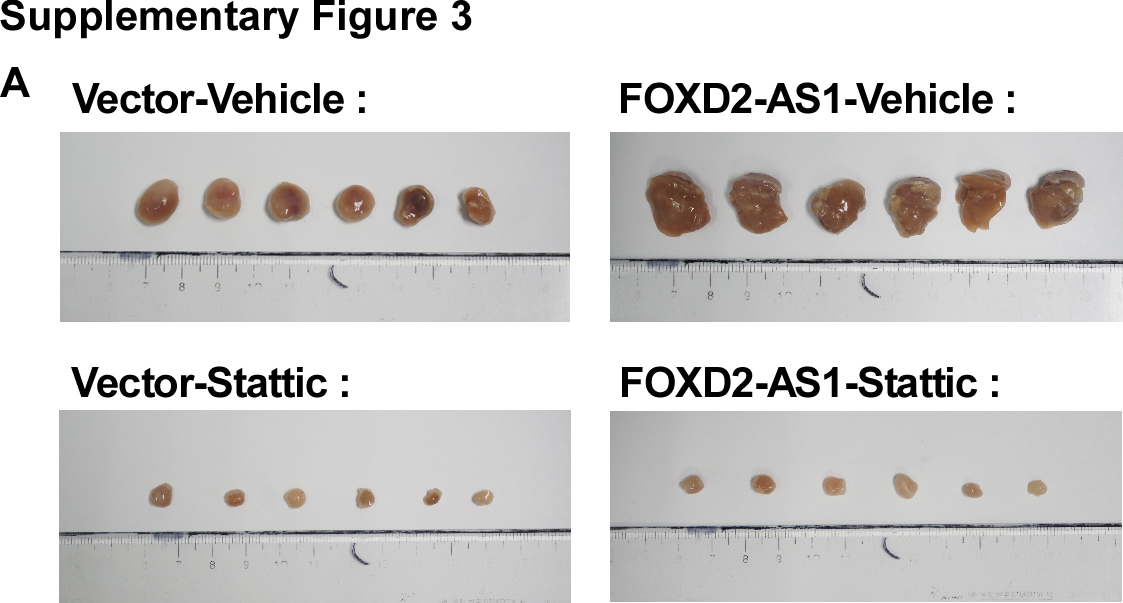

Supplement: Supplementary file 4 — Supplementary Figure 3 [file 41419_2020_2232_MOESM4_ESM.tif]
