# Supplementary material for: Long noncoding RNA FOXD2-AS1 enhances chemotherapeutic resistance of laryngeal squamous cell carcinoma via STAT3 activation
Source: Cell Death Dis. 2020 Jan 20;11(1):41. doi: 10.1038/s41419-020-2232-7 (PMC6971019; doi:10.1038/s41419-020-2232-7)
Supplement: Supplementary file 5 — Supplementary Table [file 41419_2020_2232_MOESM5_ESM.docx]

**Supplementary Table 1.** Real-time PCR primers used in this study.

| **Primer Name** | **Sequence (5'-3')** |
| --- | --- |
| MYC-F | CCTGGTGCTCCATGAGGAGAC |
| MYC-R | CAGACTCTGACCTTTTGCCAGG |
| BIRC5-F | CCACTGAGAACGAGCCAGACTT |
| BIRC5-R | GTATTACAGGCGTAAGCCACCG |
| MCL1-F | CCAAGAAAGCTGCATCGAACCAT |
| MCL1-R | CAGCACATTCCTGATGCCACCT |
| GAPDH-F | GTCTCCTCTGACTTCAACAGCG |
| GAPDH-R | ACCACCCTGTTGCTGTAGCCAA |
| POU5F1-F | CCTGAAGCAGAAGAGGATCACC |
| POU5F1-R | AAAGCGGCAGATGGTCGTTTGG |
| SOX2-F | GCTACAGCATGATGCAGGACCA |
| SOX2-R | TCTGCGAGCTGGTCATGGAGTT |
| NANOG-F | CTCCAACATCCTGAACCTCAGC |
| NANOG-R | CGTCACACCATTGCTATTCTTCG |
| FOXD2-AS1-F | CCGCGTAAGCCTCATAGAAG |
| FOXD2-AS1-R | GGGAGTAGGGTGAGGAAAGG |
