# Supplementary material for: Long noncoding RNA FOXD2-AS1 enhances chemotherapeutic resistance of laryngeal squamous cell carcinoma via STAT3 activation
Source: Cell Death Dis. 2020 Jan 20;11(1):41. doi: 10.1038/s41419-020-2232-7 (PMC6971019; doi:10.1038/s41419-020-2232-7)
Supplement: Supplementary file 7 — Supplementary [file 41419_2020_2232_MOESM7_ESM.pdf]

DECLARATION OF CONTRIBUTIONS TO ARTICLE **ADMC**

Manuscript Number:	Journal Name:	
CDDIS-19-3105R	*Cell Death & Disease*	(the 'Journal')

Proposed Title of the Contribution:

Long noncoding RNA FOXD2-AS1 enhances chemotherapeutic resistance of laryngeal squamous cell carcinoma via STAT3 activation (the 'Contribution')

Author(s):

Rui Li, Shuwei Chen, Jiandong Zhan, Xinghua Li, Wenlin Liu, Xiaoli Sheng, Zhongming Lu, Rong Zhong, Liangsi Chen, Xiaoning Luo, Yameng Hu, Ying Ouyang,Tao Liu, Quan Zhang , Siyi Zhang (the 'Authors')

For all *CDDis* articles, each person named as an author in the published version must be able to show he or she has contributed substantially to the article.

Authorship credit should be based on 1) substantial contributions to conception and design, acquisition of data, or analysis and interpretation of data; 2) drafting the article or revising it critically for important intellectual content; and 3) final approval of the version to be published. Authors should meet conditions 1, 2 and 3.

Any person who cannot be shown to have made a substantial contribution to the article cannot be listed as an author in the final version. The name of any person who is deemed to have made a minor contribution can, however, appear in the Acknowledgments section of the article.

Please complete the table below to indicate the contributions of all named authors to the manuscript.

Author Full Name:	Specification of Contribution to the Manuscript:
Rui Li,Shuwei Chen,Jiandong Zhan,Xinghua Li	Paper writing,Molecular experiment,Cell experiment,Animal experiment
Rui Li,Wenlin Liu,Xiaoli Sheng,Rong Zhong	Statistics collection
Zhongming Lu,Liangsi Chen,Xiaoning Luo	Statistics analysis
Yameng Hu, Ying Ouyang	Part of the cell experiment
Tao Liu,Quan Zhang,Siyi Zhang	Design the experiment,inspect the experiment,Statistics analysis

Please complete the table below to indicate the contributions of all named authors to the figures.

Figure 1:

These clinical samples is collected by Rui Li,Wenlin Liu,Xiaoli Sheng,Rong Zhong;These statistics are analysed by Zhongming Lu,Liangsi Chen,Xiaoning Luo,Siyi Zhang;These molecular experiments are did by Rui Li,Shuwei Chen,Jiandong Zhan,Xinghua Li;These figures are plotted by Rui Li,Shuwei Chen,Jiandong Zhan,Xinghua Li;Figure1 is designed and inspected by Siyi Zhang,Quan Zhang,Tao Liu.

Figure 2:

These statistics are analysed by Quan Zhang,Zhongming Lu,Liangsi Chen,Xiaoning Luo,Siyi Zhang;These molecular experiments and cell experiments are did by Rui Li,Shuwei Chen,Jiandong Zhan,Xinghua Li; These figures are plotted by Rui Li,Shuwei Chen,Jiandong Zhan,Xinghua Li;Figure 2 is designed and inspected by Siyi Zhang,Quan Zhang,Tao Liu.

Figure 3:

These statistics are analysed by Zhongming Lu,Liangsi Chen,Xiaoning Luo,Siyi Zhang;These molecular experiments and cell experiments, and animal experiments are did by Rui Li,Shuwei Chen,Jiandong Zhan,Xinghua Li; These figures are plotted by Rui Li,Shuwei Chen,Jiandong Zhan,Xinghua Li;Figure 3 is designed and inspected by Siyi Zhang,Quan Zhang,Tao Liu.

Figure 4:

These statistics are analysed by Zhongming Lu,Liangsi Chen,Xiaoning Luo,Siyi Zhang,Tao Liu;These molecular experiments and cell experiments are did by Rui Li,Shuwei Chen,Jiandong Zhan,Xinghua Li;Parts of these cell experiments are did by Yameng Hu, Ying Ouyang; These figures are plotted by Rui Li,Shuwei Chen,Jiandong Zhan,Xinghua Li;Figure 4 is designed and inspected by Siyi Zhang,Quan Zhang,Tao Liu.

Figure 5:

These statistics are analysised by Zhongming Lu,Liangsi Chen,Xiaoning Luo,Siyi Zhang;These molecular experiments are did by Rui Li,Shuwei Chen,Jiandong Zhan,Xinghua Li;These cell experiments are did by Rui Li,Shuwei Chen,Jiandong Zhan,Xinghua Li,Yameng Hu, Ying Ouyang; These figures are plotted by Rui Li,Shuwei Chen,Jiandong Zhan,Xinghua Li;Figure 5 is designed and inspected by Siyi Zhang,Quan Zhang,Tao Liu.

Figure 6:

These statistics are analysised by Zhongming Lu,Liangsi Chen,Xiaoning Luo,Siyi Zhang;These molecular experiments and cell experiments are did by Rui Li,Shuwei Chen,Jiandong Zhan,Xinghua Li;These figures are plotted by Rui Li,Shuwei Chen,Jiandong Zhan,Xinghua Li; Figure 6 is designed and inspected by Siyi Zhang,Quan Zhang,Tao Liu.

Signed for and on behalf of the Author(s):	Print Name:	Date:
Siyi Zhang	Siyi Zhang	2019/12/05

Rui Li, Shuwei Chen, Jiandong Zhan, Xinghua Li Ying Ouyang. Wenlin Liu xiaolisheng
Zhongming Lu Rong Zhong Liangsi Chen. Xiaoning Luo Yameng Hu Tao Liu
Quan Zhang
