# Supplementary material for: Long noncoding RNA FOXD2-AS1 enhances chemotherapeutic resistance of laryngeal squamous cell carcinoma via STAT3 activation
Source: Cell Death Dis. 2020 Jan 20;11(1):41. doi: 10.1038/s41419-020-2232-7 (PMC6971019; doi:10.1038/s41419-020-2232-7)
Supplement: Supplementary file 8 — Supplementary [file 41419_2020_2232_MOESM8_ESM.pdf]

SPRINGER NATURE

Change of authorship request form - Journals (pre-acceptance)

Important information. Please read.

- This form should be used by authors to request any change in authorship (adding/deleting authors) including changes in corresponding authors. This form should not be used for name changes. Please fully complete all sections. Use black ink and block capitals and provide each author's full name with the given name first followed by the family name.

- By signing this declaration, all authors guarantee that the order of the authors are in accordance with their scientific contribution, if applicable as different conventions apply per discipline, and that only authors have been added who made a meaningful contribution to the work.

- Please note, in author collaborations where there is formal agreement for representing the collaboration, it is sufficient for the representative or legal guarantor (usually the corresponding author) to complete and sign the Authorship Change Form on behalf of all authors, **next to the added/removed author(s). (Complete Section 3, followed by Section 6.)**
  In author collaborations where there is no formal agreement for representing the collaboration and **there are more than 10 authors**, one may sign for all, provided the signer appends correspondence that attests that each of the authors have agreed to the change **and the added/removed authors sign the form. (Complete Section 3, followed by Section 6.)**

- Please note, we cannot investigate or mediate any authorship disputes. If you are unable to obtain agreement from all authors (including those who you wish to be removed) you must refer the matter to your institution(s) for investigation. Please inform us if you need to do this.

- If you are not able to return a fully completed form within **30 days** of the date that it was sent to the author requesting the change, we may have to withdraw your manuscript. We cannot publish manuscripts where authorship has not been agreed by all authors (including those who have been removed).

- Incomplete forms will be rejected.

- Please return/upload this form, fully completed, to the Journals Editorial Office. The Journal and/or Publisher will consider the information you have provided to decide whether to approve the proposed change in authorship. We may decide to contact your institution for more information or undertake a further investigation, if appropriate, before making a final decision.

SPRINGER NATURE

Change of authorship request form - Journals (pre-acceptance)

Section 1: Please provide the current title of manuscript

Manuscript ID no.: CDDIS- 19-3015R

Title: Long noncoding RNA FOXD2-AS1 enhances chemotherapeutic resistance of laryngeal sequamous cell carcinoma via STAT3 activation

Section 2: Please provide the previous authorship, in the order shown on the manuscript before the changes were introduced. Please indicate the corresponding author by adding (CA) behind the name.

	First name(s)	Family name	ORCID or SCOPUS id, if available
1st author	Rui	Li	
2nd author	Shuwei	Chen	
3rd author	Jiandong	Zhan	
4th author	Xinghua	Li	
5th author	Wenlin	Liu	
6th author	Xiaoli	Sheng	
7th author	Zhongming	Lu	
8th author	Rong	Zhong	
9th author	Liangsi	Chen	
10th author	Xiaoning	Luo	

Please use an additional sheet if there are more than 10 authors.

SPRINGER NATURE Springer Nature is one of the world's leading global research, educational and professional publishers, created in May 2015 through the combination of Nature Publishing Group, Palgrave Macmillan, Macmillan Education and Springer Science+Business Media.

SPRINGER NATURE

Change of authorship request form - Journals (pre-acceptance)

Section 1: Please provide the current title of manuscript

Manuscript ID no.: CDDIS-19-3015R

Title: Long noncoding RNA FOXD2-AS1 enhances chemotherapeutic resistance of laryngeal sequamous cell carcinoma via STAT3 activation

Section 2: Please provide the previous authorship, in the order shown on the manuscript before the changes were introduced. Please indicate the corresponding author by adding (CA) behind the name.

	First name(s)	Family name	ORCID or SCOPUS id, if available
1st author	Tao	Liu (CA)	
2nd author	Quan	zhang (CA)	
3rd author	Siyi	zhang (CA)	https://orcid.org/0000-0001-8669-5285
4th author			
5th author			
6th author			
7th author			
8th author			
9th author			
10th author			

Please use an additional sheet if there are more than 10 authors.

SPRINGER NATURE Springer Nature is one of the world's leading global research, educational and professional publishers, created in May 2015 through the combination of Nature Publishing Group, Palgrave Macmillan, Macmillan Education and Springer Science+Business Media.

SPRINGER NATURE

Change of authorship request form - Journals (pre-acceptance)

Section 3: Please provide a justification for change. Please use this section to explain your reasons for changing the authorship of your manuscript, e.g. what necessitated the change in authorship? Please refer to the (journal) policy pages for more information about authorship. Please explain why omitted authors were not originally included and/or why authors were removed on the submitted manuscript.

These two new added authors, Yameng Hu and Ying Ouyang, made great contributions in the early experimental guidance, and also participated in most of the cell experiments. They were also fully involved in the final manuscript verification. Therefore, I would like to thank these two authors for their contributions and add them to the new author list.

Section 4: Proposed new authorship. Please provide your new authorship list in the order you would like it to appear on the manuscript. Please indicate the corresponding author by adding (CA) behind the name. <u>If the Corresponding Author has changed, please indicate the reason under section 3</u>.

	First name(s)	Family name (this name will appear in full on the final publication and will be searchable in various abstract and indexing databases)	Affiliated institute	E-mail address
1st author	Rui	Li	School of Medicine, South China University of Technology	13027737513@163.com
2nd author	Shuwei	Chen	Sun Yat-sen University Cancer Center	chenshuw@sysucc.org.cn
3rd author	Jiandong	Zhan	Guangdong Provincial People's Hospital	dizhen368cw@qq.com
4th author	Xinghua	Li	Yantai Yuhuangding Hospital	lxh1152@163.com
5th author	Wenlin	Liu	Qingyuan People's Hospital	lwlin803@126.com
6th author	Xiaoli	Sheng	Guangdong Provincial People's Hospital	shxlig@qq.com
7th author	Zhongming	Lu	Guangdong Provincial People's Hospital	entlu@126.com
8th author	Rong	Zhong	School of Medicine, South China University of Technology	zhongrft@163.com
9th author	Liangsi	Chen	Guangdong Provincial People's Hospital	lancer_chen@hotmail.com
10th author	Xiaoning	Luo	Guangdong Provincial People's Hospital	luoxn_ent@126.com

Please use an additional sheet if there are more than 10 authors.

SPRINGER NATURE Springer Nature is one of the world's leading global research, educational and professional publishers, created in May 2015 through the combination of Nature Publishing Group, Palgrave Macmillan, Macmillan Education and Springer Science+Business Media.

SPRINGER NATURE

Change of authorship request form - Journals (pre-acceptance)

Section 3: Please provide a justification for change. Please use this section to explain your reasons for changing the authorship of your manuscript, e.g. what necessitated the change in authorship? Please refer to the (journal) policy pages for more information about authorship. Please explain why omitted authors were not originally included and/or why authors were removed on the submitted manuscript.

These two new added authors, Yameng Hu and Ying Ouyang, made great contributions in the early experimental guidance, and also participated in most of the cell experiments. They were also fully involved in the final manuscript verification. Therefore, I would like to thank these two authors for their contributions and add them to the new author list.

Section 4: Proposed new authorship. Please provide your new authorship list in the order you would like it to appear on the manuscript. Please indicate the corresponding author by adding (CA) behind the name. <u>If the Corresponding Author has changed, please indicate the reason under section 3</u>.

	First name(s)	Family name (this name will appear in full on the final publication and will be searchable in various abstract and indexing databases)	Affiliated institute	E-mail address
1st author	Yameng	Hu	Zhongshan school of medicine, Sun Yat-sen University	1145892549@qq.com
2nd author	Ying	OuYang	Sun Yat-sen University Cancer Center	ouyangy1@sysucc.org.cn.
3rd author	Tao	Liu (CA)	Guangdong Provincial People's Hospital	taoliu18@126.com
4th author	Quan	Zhang (CA)	Sun Yat-sen University Cancer Center	zhangquan@sysucc.org.cn
5th author	Siyi	zhang (CA)	School of Medicine, South China University of Technology	zhangsiyi@gdph.org.cn
6th author				
7th author				
8th author				
9th author				
10th author				

Please use an additional sheet if there are more than 10 authors.

SPRINGER NATURE Springer Nature is one of the world's leading global research, educational and professional publishers, created in May 2015 through the combination of Nature Publishing Group, Palgrave Macmillan, Macmillan Education and Springer Science+Business Media.

SPRINGER NATURE

Change of authorship request form - Journals (pre-acceptance)

Section 5: Author contribution, Acknowledgement and Disclosures. Please use this section to provide a new disclosure statement and, if appropriate, acknowledge any contributors who have been removed as authors and ensure you state what contribution any new authors made (if applicable per the journal or book (series) policy). Please ensure these are updated in your manuscript - <u>after approval of the change(s)</u> - as our production department will not transfer the information in this form to your manuscript.

New acknowledgements:

There are no new acknowledgements.

New Disclosures (financial and non-financial interests, funding):

There are no new disclosures.

New Author Contributions statement (if applicable per the journal policy):

There are two new authors, Yameng Hu and Ying Ouyang. Both of them made great contributes in the early experimental guidance, and also participated in most of the cell experiments. They were also fully involved in the final manuscript verification.

State 'Not applicable' if there are no new authors.

SPRINGER NATURE Springer Nature is one of the world's leading global research, educational and professional publishers, created in May 2015 through the combination of Nature Publishing Group, Palgrave Macmillan, Macmillan Education and Springer Science+Business Media.

SPRINGER NATURE

Change of authorship request form - Journals (pre-acceptance)

Section 6: Declaration of agreement. *All* authors, unchanged, new and removed *must* sign this declaration.
(NB: Please print the form, (docu)-sign and return/upload a scanned copy. Please note that signatures that have been inserted as an image file are acceptable as long as it is handwritten. Typed names in the signature box are unacceptable.) *** Please delete as appropriate. Delete all of the bold if you were on the original authorship list and are remaining as an author.**

	First name	Family name		Signature	Date
1st author	Rui	Li	I agree to the proposed new authorship shown in section 4 **/and the addition/removal*of my name to the authorship list** /and the proposed change in corresponding author	Rui Li	2019/12/11
2nd author	Shuwei	Chen	I agree to the proposed new authorship shown in section 4 **/and the addition/removal*of my name to the authorship list** /and the proposed change in corresponding author	Shuwei Chen	2019/12/11
3rd author	Jiandong	Zhan	I agree to the proposed new authorship shown in section 4 **/and the addition/removal*of my name to the authorship list** /and the proposed change in corresponding author	Jiandong Zhao	2019/12/11
4th authors	Xinghua	Li	I agree to the proposed new authorship shown in section 4 **/and the addition/removal*of my name to the authorship list** /and the proposed change in corresponding author	Xinghua Li	2019/12/11
5th author	Wenlin	Liu	I agree to the proposed new authorship shown in section 4 **/and the addition/removal*of my name to the authorship list** /and the proposed change in corresponding author	Wenlin Liu	2019/12/11
6th author	Xiaoli	Sheng	I agree to the proposed new authorship shown in section 4 **/and the addition/removal*of my name to the authorship list** /and the proposed change in corresponding author	Xiaoli Sheng	2019/12/11
7th author	Zhongming	Lu	I agree to the proposed new authorship shown in section 4 **/and the addition/removal*of my name to the authorship list** /and the proposed change in corresponding author	Zhongming Lu	2019/12/11

SPRINGER NATURE Springer Nature is one of the world's leading global research, educational and professional publishers, created in May 2015 through the combination of Nature Publishing Group, Palgrave Macmillan, Macmillan Education and Springer Science+Business Media.

Change of authorship request form - Journals (pre-acceptance)

	First name	Family name		Signature	Date
8th author	Rong	Zhong	I agree to the proposed new authorship shown in section 4 **/and the addition/removal*of my name to the authorship list** /and the proposed change in corresponding author	Rong Zhong	2019.12.11
9th author	Liangsi	Chen	I agree to the proposed new authorship shown in section 4 **/and the addition/removal*of my name to the authorship list** /and the proposed change in corresponding author	Liangsi Chen	2019. 12.11
10th author	Xiao ning	Luo	I agree to the proposed new authorship shown in section 4 **/and the addition/removal*of my name to the authorship list** /and the proposed change in corresponding author	Liaoning Luo	2019.12.11

Please use an additional sheet if there are more than 10 authors.

In case of author collaborations with formal agreement:

	Name of consortium/consortia	First name	Family name		Signature	Date
Representative/legal guarantor				I agree to the proposed new authorship shown in section 4 **/and the addition/removal*of my name to the authorship list** /and the proposed change in corresponding author		

Both added/removed authors should complete the information in the first table under Section 6.

---- End of form ----

SPRINGER NATURE Springer Nature is one of the world's leading global research, educational and professional publishers, created in May 2015 through the combination of Nature Publishing Group, Palgrave Macmillan, Macmillan Education and Springer Science+Business Media.

SPRINGER NATURE

Change of authorship request form - Journals (pre-acceptance)

Section 6: Declaration of agreement. *All* authors, unchanged, new and removed *must* sign this declaration.
(NB: Please print the form, (docu)-sign and return/upload a scanned copy. Please note that signatures that have been inserted as an image file are acceptable as long as it is handwritten. Typed names in the signature box are unacceptable.) *** Please delete as appropriate. Delete all of the bold if you were on the original authorship list and are remaining as an author.**

	First name	Family name		Signature	Date
1st author	Yameng	Hu	I agree to the proposed new authorship shown in section 4 **/and the addition/removal*of my name to the authorship list** /and the proposed change in corresponding author	Yameng Hu	2019.12.11
2nd author	Ying	Ouyang	I agree to the proposed new authorship shown in section 4 **/and the addition/removal*of my name to the authorship list** /and the proposed change in corresponding author	Ying Ouyang	2019.12.11
3rd author	Tao	Liu	I agree to the proposed new authorship shown in section 4 **/and the addition/removal*of my name to the authorship list** /and the proposed change in corresponding author	Tao Liu	2019.12.11
4th authors	Quan	Zhang	I agree to the proposed new authorship shown in section 4 **/and the addition/removal*of my name to the authorship list** /and the proposed change in corresponding author	Quan Zhang	2019.12.11
5th author	Siyi	Zhang	I agree to the proposed new authorship shown in section 4 **/and the addition/removal*of my name to the authorship list** /and the proposed change in corresponding author	Siyi Zhang	2019.12.11
6th author			I agree to the proposed new authorship shown in section 4 **/and the addition/removal*of my name to the authorship list** /and the proposed change in corresponding author		
7th author			I agree to the proposed new authorship shown in section 4 **/and the addition/removal*of my name to the authorship list** /and the proposed change in corresponding author		

SPRINGER NATURE Springer Nature is one of the world's leading global research, educational and professional publishers, created in May 2015 through the combination of Nature Publishing Group, Palgrave Macmillan, Macmillan Education and Springer Science+Business Media.
